# Advancing digital health literacy in cancer care: Recommendations from two nominal group technique workshops in the TRANSiTION project

**DOI:** 10.12688/openreseurope.21439.4

**Published:** 2026-05-21

**Authors:** David Liñares, Noemí López-Rey, Andreas Charalambous, Constantina Cloconi, Dimitrios Protogiros, Efthyvoulos Kyriacou, Iolie Nicolaidou, Nikolina Dodlek, Theologia Tsitsi, Álvaro Jimber, Norbert Couespel, Ana Clavería

**Affiliations:** 1 Avalia-t, Galician Agency for Health Technology Assessment, Santiago de Compostela, Galicia, Spain; 2 I-Saúde Group, Galicia Sur Health Research Institute, Vigo, Galicia, Spain; 3 Research Network in Chronicity, Primary Care and Health Promotion, Vigo, Galicia, Spain; 4 A Milagrosa Health Center, Servicio Gallego de Salud, Lugo, Galicia, Spain; 5 Cyprus University of Technology, Limassol, Cyprus; 6 University of Turku, Turku, Finland; 7 European Cancer Organisation, Brussels, Belgium

**Keywords:** Neoplasms, Digital Health, Health Literacy, Health Personnel, Professional Competence, Telemedicine, Health Equity

## Abstract

**Background:**

Cancer is a major cause of mortality and morbidity in Europe, with increasing focus on integrating digital health tools to ensure safe, patient-centred, and equitable oncology care. However, gaps in digital health literacy among healthcare professionals limit the effective use of these innovations. In response to these challenges, the TRANSiTION project was launched to develop a comprehensive training framework in digital health literacy for the oncology workforce. Within this framework, the present study aimed to identify and prioritise the digital health literacy training needs of clinical and non-clinical cancer care professionals across Europe.

**Methods:**

This qualitative study used the Nominal Group Technique (NGT) within two user experience (UX) design workshops. A total of 44 professionals participated, purposively sampled to capture diverse expertise in oncology and digital health. The first workshop (n = 34) was composed predominantly of non-clinical professionals, while the second (n = 10) involved mainly clinical professionals directly engaged in cancer care. Recommendations generated during the workshops were analysed descriptively, and participant contributions were consolidated in real time to support the identification and prioritisation of training needs.

**Results:**

Across both workshops, five shared priorities were highlighted: (1) ethics, particularly data protection, trust, and responsible use of digital tools; (2) integration of telemedicine to empower patients, provided it fosters safety and confidence; (3) development of user-friendly, accessible, and adaptable training interfaces; (4) addressing the digital divide, especially for rural populations, older adults, and low-resource regions; and (5) continuous training for cancer professionals, with formal recognition of digital skills as core competencies.

**Conclusions:**

This study identified actionable priorities to guide the development of a digital health literacy training framework for oncology professionals. Embedding ethics, usability, equity, and continuous training into professional development can strengthen workforce readiness, enhance patient empowerment, and improve outcomes across Europe. These findings provide a foundation for the TRANSiTION framework and for future strategies to integrate digital health literacy into cancer care.

## Plain Language Summary

Cancer is one of the most common and serious health challenges in Europe. Although many digital tools (such as telemedicine, mobile apps, and electronic health records) are increasingly used in cancer care, many healthcare professionals lack the digital skills needed to use them effectively. This includes those who provide direct patient care (known as clinical professionals) as well as those in administrative or support roles (non-clinical professionals).

Our study, part of the TRANSiTION project, set out to identify and prioritise the digital training needs of both groups across Europe. We held two workshops with 44 participants from various countries: one mostly comprised non-clinical professionals, and the other mainly clinical professionals. Participants discussed and voted on the most important digital literacy needs.

From these discussions, five key priorities emerged:

1. Ensuring ethical and trustworthy use of digital tools, including data protection and privacy;
2. Using telemedicine to empower patients, provided it feels safe and reliable;
3. Designing training platforms that are user-friendly, adaptable, and accessible;
4. Addressing digital inequalities, particularly for rural populations, older adults, and low-resource communities;
5. Offering ongoing training for all professionals, with formal recognition of digital skills as core competencies.

This study provides a roadmap for creating training programs that enable health professionals to use digital tools effectively and equitably, helping to improve cancer care across Europe.

## Introduction

Cancer remains the second leading cause of premature death and disability worldwide ( Bray *et al.*, 2018). In the European Union, it accounts for approximately 4.7 million new cases and 2.1 million deaths annually ( Dyba *et al.*, 2021). Recognising the magnitude of this public health challenge, the European Commission (EC) has prioritised cancer control as a key policy objective. This is reflected in Europe’s Beating Cancer Plan (EC, 2022), which outlines strategic actions to improve cancer prevention, early detection, treatment, and survivorship.

Cancer care involves long-term follow-up, complex treatment pathways, and high volumes of medical information, which demand that patients and professionals make informed decisions continuously throughout the care trajectory ( Shickh *et al.*, 2023). In this context, the digital transformation of healthcare is reshaping how cancer care is delivered and experienced. Telemedicine, electronic health records, mobile apps, and artificial intelligence tools are becoming increasingly embedded in oncology practice. However, capitalising on these advances requires adequate digital health literacy among both professionals ( Tuominen *et al.*, 2024b). Simultaneously, patients, particularly older adults, individuals living in rural areas, or those with lower socioeconomic status, often face substantial barriers to digital health access and engagement, including poor internet infrastructure, limited skills, and lack of tailored support ( Gottlob *et al.*, 2025; Yuen *et al.*, 2024). These disparities highlight the urgent need to incorporate digital health literacy as a fundamental component of cancer care strategies, to ensure equitable access and improved health outcomes for all.

Despite the increasing presence of digital tools in oncology, structured training in digital health literacy for healthcare professionals remains limited ( Liñares & Charalambous, 2025; Protogiros *et al.*, 2025). Existing educational programs often fail to address the full spectrum of digital competencies needed to safely and effectively support patients in digital environments ( Tischendorf *et al.*, 2024). Moreover, the implementation of digital health solutions raises ethical, legal, technical, and policy-related challenges such as data privacy, digital equity, and the regulation of telemedicine ( Livieri *et al.*, 2025). A key limitation is the absence of consensus on what digital health literacy competencies should be included in professional development frameworks, which hampers the development of coherent, actionable, and widely applicable training strategies ( Lawrence & Levine, 2024). Furthermore, training needs in cancer care vary depending on professional roles, with clinical and non-clinical professionals requiring different sets of digital competencies to fulfil their respective functions effectively ( Liñares *et al.*, 2025). Addressing these gaps is essential to ensure that healthcare professionals are equipped to meet the demands of digital cancer care.

In view of these challenges, the TRANSiTION project was co-funded by the European Union to address the digital training needs of the oncology workforce ( https://www.europeancancer.org/hpv/impact/resource/transition.html). The project aims to develop an advanced training programme for both clinical and non-clinical professionals involved in cancer care, equipping them with the digital competencies needed to enhance the effectiveness and efficiency of information exchange with patients and other healthcare stakeholders. TRANSiTION brings together an interdisciplinary consortium of 24 partners across 14 EU Member States, each with extensive expertise in designing, evaluating, and implementing continuing professional development programmes in oncology. As part of this initiative, one of the project’s work packages focused on assessing training needs, with the objective of identifying both the specific requirements of future users and existing gaps in digital health literacy among professionals working in cancer care. Specifically, it responds to the lack of consensus on digital health literacy competencies and seeks to establish a coherent training framework.

Building on this rationale, the Nominal Group Technique (NGT) provides a structured, transparent process that elicits diverse perspectives, mitigates dominance effects, and yields ranked priorities suited to decision-making and guideline development in healthcare ( Allen *et al.*, 2004; McMillan *et al.*, 2016). Recent methodological work also supports virtual adaptations of NGT when appropriate ( Lee *et al.*, 2024), and offers guidance for rigorous reporting ( Gattrell *et al.*, 2024). In oncology, NGT has been used to prioritize evidence–practice gaps in lung cancer care ( Rankin *et al.*, 2016), define quality metrics in pediatric end-of-life cancer care ( Johnston *et al.*, 2024), and develop, refine, and prioritize patient-care recommendations and service priorities across settings ( Algeo *et al.*, 2022; Humphrey *et al.*, 2025; Somers *et al.*, 2019; Stiger *et al.*, 2025).

Accordingly, this study reports on two user experience (UX) design workshops conducted within the TRANSiTION project, each applying the NGT to elicit expert input on the digital training needs of cancer care professionals. The aim was to generate actionable, prioritised recommendations to inform the development of a comprehensive training framework, capturing perspectives from both clinical and non-clinical professionals across diverse countries and healthcare settings.

## Method

### Design

This study adopted a qualitative descriptive design underpinned by the NGT. It was implemented through two UX design workshops as part of the TRANSiTION project (Grant Agreement No. 101101261), which aims to develop a comprehensive training framework to enhance digital health literacy among clinical professionals (CP) and non-clinical professionals (NCP) involved in cancer care. CP are defined as members of healthcare organisations providing direct cancer care (e.g., oncologists, radiotherapists, oncology nurses, family physicians, and community nurses), whereas NCP refers to individuals performing administrative, managerial, or support functions related to cancer care, regardless of their professional background.

Within the TRANSiTION project, these workshops were conceived as an initial needs assessment activity to inform the training framework design and to generate actionable recommendations for partners and stakeholders. A user experience (UX) design approach was used to structure the sessions and support the elicitation of practical requirements for digital health literacy training. The NGT was selected because it provides a structured consensus process, supports balanced participation, and produces ranked priorities suitable for decision making and framework development. In this study, UX provided the setting and materials for idea generation and consolidation, while NGT provided the stepwise procedure for producing and prioritising recommendations.

### Participants

Two UX design workshops were conducted. Participants were selected through purposive sampling to ensure the inclusion of individuals with relevant expertise in cancer care and digital health literacy. Recruitment was carried out by members of the TRANSiTION consortium, who shared the workshop invitations via email and identified potential participants who met the predefined inclusion criteria. Inclusion criteria were: (a) current professional involvement in cancer care, either in a clinical or non-clinical role, and (b) active engagement in their professional position at the time of the study. No minimum years of professional experience were required. Consortium members were also eligible to participate. Therefore, some participants were members of the TRANSiTION consortium or had prior professional links with the research team, whereas others were external invitees with no previous relationship with the facilitators. These prior links may have meant that some participants were more familiar with the aims, terminology, and expected outputs of the project before the workshops.

### Procedure

Each workshop was guided by a central focus question presented to all participants: *“Considering the existing needs, select an ethical, policy, legal and/or technical recommendation, to partners or stakeholders, to ensure digital health literacy in cancer care.”* This question was formulated within the framework of the TRANSiTION project, reflecting its emphasis on the ethical, policy, legal, and technical dimensions of digital health literacy in cancer care. It served as the basis for the idea generation and voting process. No pilot testing was conducted.

The first workshop was held on 25 September 2023 in a hybrid format, combining in-person and online participation, in Limassol, Cyprus. The second took place on 21 October 2023 in Madrid, Spain, and was conducted entirely in person.

Both workshops followed the same structured format based on the NGT. The first session lasted 120 minutes, beginning with 25 minutes dedicated to background information and participant introductions. This was followed by a 90-minute NGT session. During this time, each participant had 2 minutes to silently write up to two recommendations. Participants then shared their suggestions aloud, one at a time, without elaboration. Once all ideas were presented, a group discussion was held to clarify and consolidate the proposals, merging similar suggestions into unified statements. Participants subsequently voted for the three recommendations they considered most important. The final 5 minutes of the workshop were reserved for acknowledgements and closing remarks.

The second workshop followed the same structure, but with a shorter total duration of 90 minutes: 25 minutes for the background presentation, 60 minutes for the NGT session, and 5 minutes for the closing. The time reduction reflected the smaller group size.

Both workshops were conducted in English to ensure consistent communication among participants from diverse countries. As the TRANSiTION consortium included members from across Europe and beyond, English served as the shared working language, supporting inclusive participation and alignment with the project’s dissemination and communication strategy.

Each session was led by two members of the research team, both full-time researchers and members of the TRANSiTION consortium. One was a female physician specialised in public health with a PhD; the other was a male psychologist with a PhD in consumer and user research. Both had prior training and experience in the application of qualitative methods, including structured group techniques. One served as the main moderator, introducing the session, coordinating speaking turns, and guiding the consolidation of ideas. The second researcher conducted a real-time consolidation of participant contributions and took supporting notes to facilitate the preparation of consolidated recommendations for the voting phase. All participants received an information sheet in advance, outlining the study’s objectives, the researchers’ roles, and the intended use of the findings to support digital health literacy in cancer care. Given that some participants had prior links with the TRANSiTION consortium, the facilitation process was structured to reduce the potential influence of pre-existing relationships on group discussion and prioritisation. The NGT procedure included silent individual idea generation, sequential sharing of suggestions, group clarification of overlapping ideas, and anonymous voting. These steps were intended to support balanced participation and ensure that final priorities reflected the structured voting process rather than the views of specific participants or consortium roles.

Each session was video recorded with participants’ consent for documentation and analysis purposes. No repeat workshops or follow-up interviews were conducted. Transcripts were not returned to participants, as data collection was based on structured prompts, digital input, and real-time group discussion. Data saturation was not applicable given the consensus-oriented nature of the methodology.

Informed consent for participation was obtained verbally from all participants prior to the workshops. Verbal consent was considered appropriate given the time-limited, workshop-based format (hybrid and in-person) and the minimal-risk nature of the activity. Consent was explicitly requested and audio-visually recorded at the beginning of each session, including consent for video recording for documentation and analysis purposes. Written consent was therefore not collected. Ethics approval/waiver was confirmed by the Pontevedra–Vigo–Ourense Research Ethics Committee (ref: 2023/309).

### Data analysis

A descriptive univariate analysis was conducted using data collected via the Slido.com platform during both UX design workshops. No formal refusals or dropouts were reported, and all individuals who agreed to participate completed the session. The analysis focused on the frequency of responses and prioritisation scores assigned during the voting phase of the NGT. The most frequently selected recommendations were identified and ranked by the number of votes received. No inferential statistical tests were applied, as the goal was to identify areas of consensus and priority among expert participants rather than to generalise findings.

Additionally, a real-time thematic consolidation of participant contributions was conducted by one researcher during each session to prepare the list of recommendations for the voting phase. This process involved grouping overlapping or similar suggestions into consolidated statements that could be discussed, refined, and prioritised by participants. When uncertainty arose regarding the grouping or interpretation of specific ideas, the facilitator consulted the participants to confirm the appropriateness of the categorisation, and the final consolidated list was agreed upon by the group before proceeding to the voting phase. This process was part of the structured NGT procedure and was intended to support voting, rather than to produce a full qualitative thematic analysis. Therefore, no formal coding tree was created, no qualitative analysis software was used, and no post hoc transcript-based coding or interpretive thematic analysis was conducted. Although sessions were recorded for documentation and analysis purposes, the recordings were not used to conduct a full transcript-based qualitative analysis. The reported themes reflect the real-time consolidation of participant contributions and the descriptive summary of voting results.

## Results

In addition to the prioritisation outcomes obtained through voting, the structured discussion and real-time consolidation conducted during the NGT sessions allowed the identification of recurring themes across participant contributions. These themes emerged during the consolidation of recommendations and reflect shared concerns and priorities expressed by participants. The themes are presented below to provide contextual understanding of how recommendations were grouped and prioritised within each workshop.

### First UX design workshop

The first UX design workshop involved 34 participants (25 women, 9 men) from organisations based in 16 countries: Belgium, Bulgaria, Croatia, Cyprus, Czech Republic, France, Germany, Hungary, Latvia, Portugal, Romania, Saudi Arabia, Slovenia, Spain, Switzerland, and the United States. Participants were affiliated with institutions such as the Institute of Information Theory and Automation of the CAS, Institut Català d’Oncologia (ICO), International Systems Engineering (ISE), Riga Stradins University (RSU), The Oncology Institute “Prof. Dr. Ion Chiricuţă” Cluj-Napoca, Universitat Oberta de Catalunya (UOC), University of Bern (UB), University of Caen Normandy (UCN), University of Cyprus (UC), University of Lorraine, University of Maribor (UM), University of New Mexico (UNM), University of Osijek (UNIOS), and University of the Balearic Islands (UIB). Of the total sample, 25 participants attended in person, and 9 participated online. Among them, 3 were CP and 31 were NCP.

Given that most participants were NCP, the session allowed for in-depth reflection from this perspective. During the group discussion, the consolidated recommendations (see Table 1) were reviewed and prioritised. The topic that received the most attention was the design and delivery of digital literacy training resulting from the TRANSiTION project. Participants stressed the importance of combining accurate and up-to-date content with a user-friendly interface tailored to the resources available. They agreed that “excellent content is of little use if it fails to engage the end users.”

**Table 1.** Feedback from the first UX design workshop.

***Considering the existing needs, select an ethical, policy, legal and/or technical recommendation, to partners or stakeholders, to ensure digital health literacy in cancer care***
“ *Technical - Prioritize accessibility features, such as text-to-speech and screen readers, to ensure that individuals with disabilities can effectively use these tools.”* *“Include digital health courses for HCPs into national training programs.”* *“Technical aspects should be very simple, intuitive, structured, and not overwhelming with information, with options to personalise the content/menu. Legal: all basic legal aspects related to regular workflow.”* *“Empower patients to use e.g., online portals and telehealth services.”* *“Increase in ICT related workforce in healthcare to support healthcare professionals. Training, funding, policy, organisational HR strategy, etc.”* *“Educating healthcare professionals to be more positive towards change and to accept and adapt to new technologies.”* *“We have to ensure the education of health care providers on digital tools.”* *“New policies and procedures for telemedicine in health care. Especially in cancer care and in rural areas. New protocols and guidelines for ethical matters for the digital care is needed.”* *“Technical.”* *“Use common data models and translations.”* *“Collaboration between non-clinicians and clinicians.”* *“Communication with the cancer patients through constant feedback.”* *“Understand the potential benefits of digital health; updated and continuous training for professionals.”* *“Ethics especially in AI in healthcare.”* *“Protection of personal information, Implementation within the NHS.”*

During the group discussion and consolidation phase, several recurring themes emerged from participant contributions. Beyond training design and usability, telemedicine emerged as a prominent theme, particularly in relation to patient empowerment and improved access to care, provided that digital tools promote safety and confidence among users. Ethical considerations were primarily framed around data protection, responsible use of digital technologies, and the need for clear legal and organisational guidance. Participants also emphasised the importance of addressing the digital divide, especially in rural areas and resource-limited settings, as a necessary condition for equitable implementation of digital health solutions.

In line with the NGT, the ideas raised during the discussion were consolidated and presented for prioritisation through voting. Participants identified several key recommendations to guide the development of the TRANSiTION training framework. A total of 102 votes were obtained from the 34 participants.

**Figure 1.**
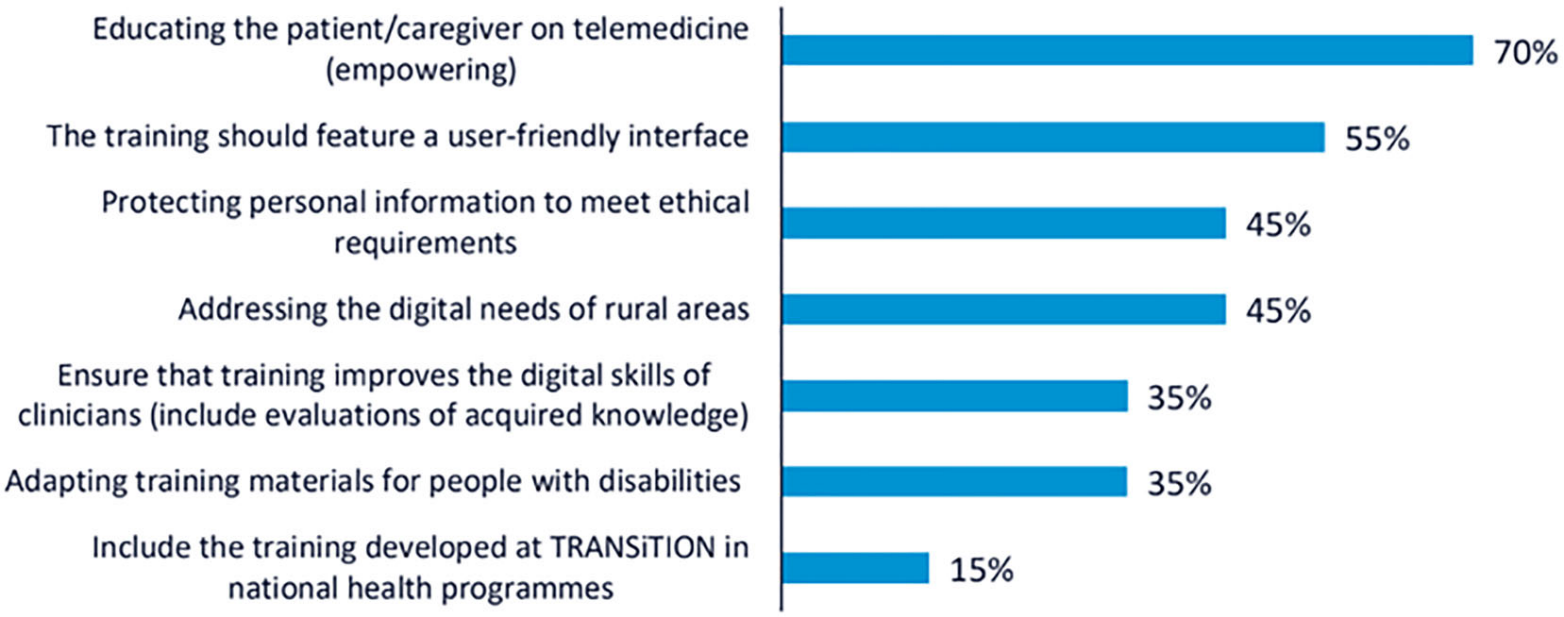
Percentage of participants selecting each recommendation during the voting phase of the first UX design workshop.

The most highly prioritised recommendation was to promote telemedicine as a central component of training, with the aim of increasing patient empowerment in cancer care settings. This recommendation received 24 votes, with approximately 70% of participants choosing it. The second most voted recommendation, selected by 19 participants (55%), emphasised the importance of developing a user-friendly interface for the training platform. Participants noted that the effectiveness of the training depends not only on content quality but also on usability and accessibility for diverse user profiles. Two recommendations received equal support and were jointly ranked third (n = 15; 45%): the need to train both CP and NCP in the ethical and secure handling of personal data, and the importance of addressing the specific challenges of rural areas, particularly by reducing the digital divide and ensuring equal access to digital resources. These results illustrate the participants’ shared concern for ethical, technical, and contextual dimensions of digital health literacy in cancer care. The rest of the recommendations collected are shown in Figure 1.

### Second UX design workshop

The second workshop included 10 participants (6 women, 4 men), aged between 29 and 48 years, from 6 countries: Belgium, Cyprus, Greece, Portugal, Spain, and Turkey. Participants were affiliated with the following organisations: Associação de Investigação de Cuidados de Suporte em Oncologia (AICSO), Cyprus University of Technology (CUT), European Cancer Organisation (ECO), European Oncology Nursing Society (EONS), Hospital Universitario Infanta Sofía, Koc University, and Servizo Galego de Saúde (SERGAS). Of these, 6 were CP and 4 were NCP.

The second UX design workshop followed the same structured procedure as the first. In this session, the composition of the group included a higher proportion of CP. This sampling strategy was intentional, aimed at capturing deeper insights and reflections from those directly involved in patient care.

The participants’ contributions, collected via the Slido.com platform, reflected a strong concern for ethical considerations in the digital transformation of healthcare (see Table 2). The most extensively discussed topic during the group debate was the role of ethics in the computerisation of medicine. Participants raised questions about how the TRANSiTION project could uphold the ethical rights of both professionals and patients in the shift toward digital health solutions.

**Table 2.** Feedback from the second UX design workshop.

***Considering the existing needs, select an ethical, policy, legal and/or technical recommendation, to partners or stakeholders, to ensure digital health literacy in cancer care***
*“Combination of digital skills education for clinical professionals with specific training on data security”* *“Ensure the safety of data (ethical); ensure the quality of applications and regulate its use -quality and loyalty assurance (legal); development with the input by users and updated continuously (technical)”* *“I would say there needs to be a combination approach between policy (digital skills being part of professional qualification requirements) and technical (clinical training) intervention to ensure health literacy and its sustainability”* *“Depending on what exactly digital health literacy obtains within Transition project, I would always first address the ethical and legal side towards the patient prior technical implementation itself”* *“Ethical & technical followed by policy”* *“Policy recommendation: supporting local community efforts on digital literacy for professionals from areas and population groups affected by the digital divide (e.g., Eastern Europe, rural areas, older generations, etc.)”* *“If I have to choose then I will choose the ethical and technical part”*

During the discussion and consolidation phase, several recurring themes emerged from participant contributions. Ethical considerations represented the dominant theme, with participants expressing concerns regarding data security, responsible implementation of digital tools, and the need to preserve patient trust in increasingly digitalised care environments. The importance of fostering a sense of safety and confidence among patients when using digital solutions was repeatedly emphasised.

Another recurring theme concerned the need to adapt digital health training to patient preferences and levels of digital readiness, highlighting the role of professionals in supporting shared decision-making when introducing digital tools into care pathways. Participants also stressed the relevance of addressing digital inequalities affecting rural populations and older adults, as well as the importance of recognising digital competencies as a core component of professional qualification and career development.

A total of 30 votes were obtained from the 10 participants. The top-ranked recommendation, supported by 8 participants (80%), was to provide training tailored to individual patient preferences. This implies that both CP and NCP should be trained to assess patients’ digital readiness and incorporate digital solutions into care in a way that promotes shared decision-making.

As in the first workshop, 4 participants (40%) highlighted the importance of addressing the specific needs of rural populations and older adults. Participants stressed that the training should consider the digital divide and disparities in access to technological resources across regions and demographic groups.

**Figure 2.**
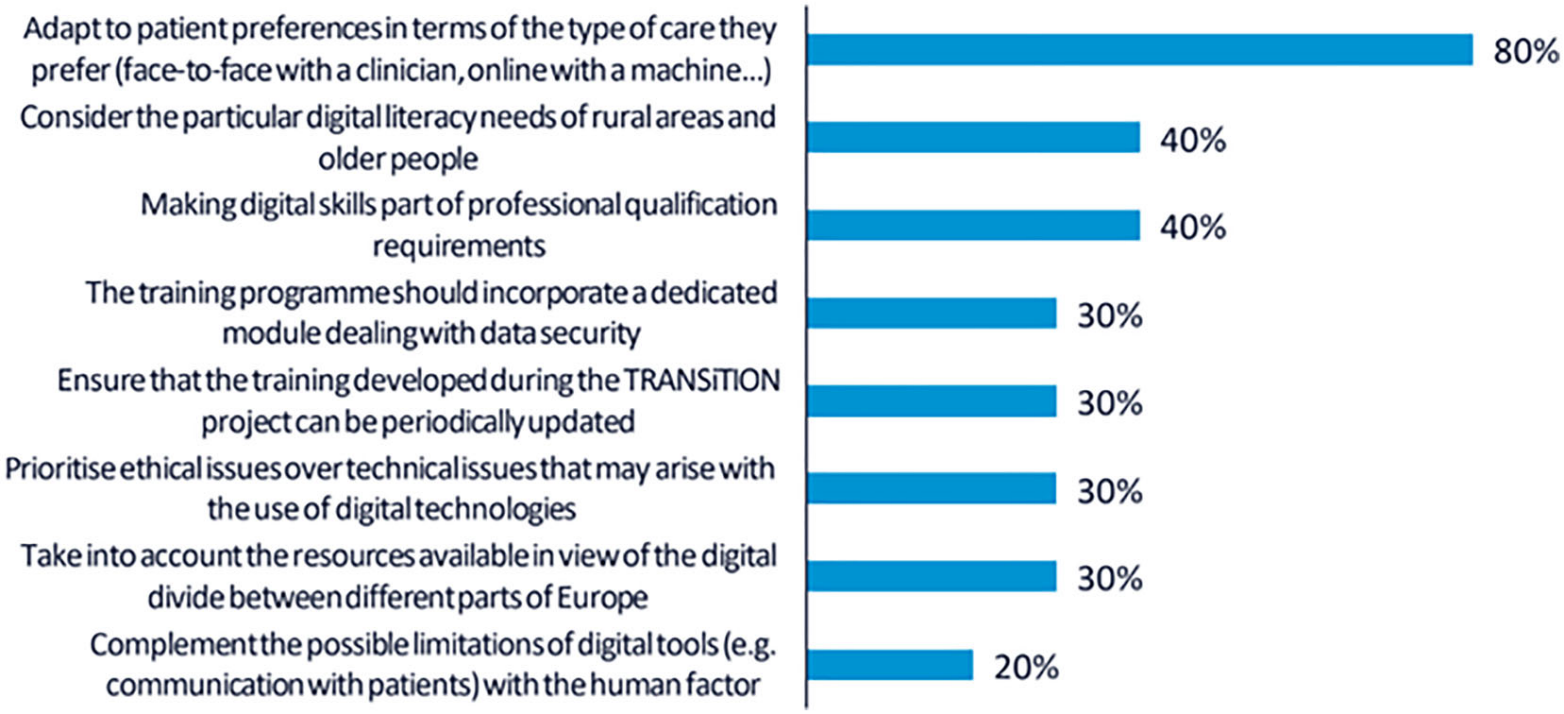
Percentage of participants selecting each recommendation during the voting phase of the second UX design workshop.

Another 4 participants (40%) emphasised the relevance of formally recognising digital skills as a core component of professional competence. In this regard, it was recommended that the training credentials offered through the TRANSiTION project should be meaningful for career development and valued within the professional trajectories of both clinical and non-clinical staff. The rest of the recommendations collected are shown in Figure 2.

When comparing the outcomes of both workshops, some differences in emphasis were observed. These differences should be interpreted cautiously, as the workshops differed not only in participant composition, but also in size and format. The first workshop, which was larger, hybrid, and composed predominantly of NCP, appeared to place greater emphasis on technical design and implementation aspects of digital health literacy training, including usability and the integration of telemedicine. The second workshop, which was smaller, conducted in person, and involved a higher proportion of CP, appeared to give more prominence to ethical considerations, patient trust and confidence in digital interactions, and the recognition of digital competencies as a core component of professional qualification. Therefore, these differences should be understood as descriptive patterns within the two workshops rather than as direct comparisons between professional groups.

## Discussion

### Main results

The two UX design workshops, conducted using the Nominal Group Technique (NGT), identified five main priorities for advancing digital health literacy in oncology care: (1) ethics emerged as a cross-cutting concern, particularly data protection, trust, and the responsible use of digital tools; (2) telemedicine was highlighted as a key strategy to empower patients, provided it fosters a sense of safety and confidence; (3) participants emphasised the need for user-friendly, accessible, and adaptable interfaces, with training tailored to patients’ preferences and digital readiness; (4) both workshops stressed the importance of addressing the digital divide to ensure equity, especially in rural areas, older populations, and regions with fewer resources; and (5) continuous training for both CP and NCP was seen as essential, with calls to formally recognise digital skills as a core professional competence.

Despite this overall convergence in priorities, some differences in emphasis were observed between workshops. These differences should be interpreted with caution because the two workshops differed in size, format, and participant composition. The first workshop was larger, hybrid, and included predominantly NCP, whereas the second was smaller, conducted in person, and involved a higher proportion of CP. Therefore, the observed differences should be considered descriptive patterns emerging from each workshop context, rather than evidence of systematic differences between CP and NCP. With this caution in mind, the first workshop appeared to place greater emphasis on technical design, usability, and telemedicine, whereas the second gave more prominence to ethical considerations, patient trust, and the formal recognition of digital skills.

### Comparison with other studies

The findings of this study are broadly consistent with previous research highlighting ethics, usability, equity, and professional training as central dimensions of digital health. In the context of an ongoing infodemic, healthcare users increasingly struggle to identify accurate and reliable information and to place trust in both the content they receive and the communication channels through which it is delivered ( Eysenbach, 2020). It is therefore unsurprising that participants voiced increasing concerns regarding data protection, trust, and the responsible use of emerging technologies, reflecting longstanding calls in the literature for ethical governance and transparency in digital health and artificial intelligence, including in oncology care ( Bouderhem, 2024; Grosman-Rimon & Wegier, 2024; Tilala *et al.*, 2024).

Some authors have warned that telemedicine can be regarded as a double-edged sword ( Petretto *et al.*, 2024). While it has the potential to expand access to treatment, socioeconomically and clinically vulnerable populations—those who might benefit the most—are often the least likely or able to use it. In cancer care, the prioritisation of telemedicine as a strategy to empower patients aligns with previous evidence highlighting its contribution to improving accessibility and satisfaction ( Sirintrapun & Lopez, 2018; West *et al.*, 2022). However, our results add that such empowerment requires professionals to foster a sense of safety and trust, and that digital solutions should be implemented in accordance with patients’ preferences, as imposing non-accepted tools risks undermining engagement. This interpretation is consistent with the work of Huret *et al.* (2022), who identified three eligibility criteria for telemedicine consultations: they should not be used for first visits, the patient should be well known to the physician and trusted to provide an accurate description of symptoms, and recent evaluations and test results should be satisfactory.

Telemedicine has been identified as a potential pathway to mitigate health inequities, with studies showing that remote consultation can improve access to specialist care in rural emergency contexts, and that its role in oncology could be strengthened when supported by adequate infrastructure and policy frameworks ( Elder *et al.*, 2023; Tsou *et al.*, 2021). Nevertheless, both workshops stressed the importance of addressing the digital divide, particularly in rural areas and among older populations, as a prerequisite for advancing equitable digital cancer care. The workshops also underscored differences across European countries, which mirror broader inequalities in digital infrastructure and health system readiness. This concern is consistent with evidence indicating that rural oncology patients face persistent challenges in accessing and effectively using digital health technologies ( Morris *et al.*, 2022). Furthermore, cancer survivors living in rural areas tend to exhibit significantly lower levels of e-health literacy, which impairs their ability to identify, interpret, and apply online health information ( Yuen *et al.*, 2024). Addressing these gaps requires not only investment in infrastructure but also tailored training and inclusive design strategies that ensure digital health innovations are accessible to all patients.

In this sense, professional training in digital health literacy should be regarded not only as a competence requirement but also as a strategic lever to mitigate inequities in access and outcomes. To achieve this, both CP and NCP must be equipped with the digital competencies needed to recognise and respond to these disparities. The call for continuous training in digital skills reflects the position of the scientific community and healthcare decision-makers, who increasingly acknowledge digital competencies as core elements of healthcare practice ( Charalambous, 2024; Longhini *et al.*, 2022).

Finally, NCP emphasised that digital skills training programmes in oncology should incorporate a user-friendly interface. While digital literacy initiatives often focus on content and conceptual frameworks, less attention is paid to how information is presented and how engaging it is for end users. The importance of UX in digital health literacy training is well established. UX research highlights the need to understand users, their contexts, and their expectations, as these factors determine whether digital innovations are accepted and adopted ( Gilbert, 2022). In the specific context of eHealth tools, usability, accessibility, reliability, and overall user experience are essential to ensure effectiveness and inclusivity ( Shamsujjoha *et al.*, 2021). Studies on digital readiness among healthcare professionals further demonstrate that perceived ease of use and organisational support—key UX elements—are critical for technology acceptance, while adequate training and facilitating infrastructure help overcome digital barriers ( Alotaibi *et al.*, 2025). This is also consistent with broader research on usability in eHealth, which shows that simplicity and adaptability to patient needs are decisive factors for adoption ( Broekhuis *et al.*, 2021; Maramba *et al.*, 2019).

### Impact in organizations and health

The findings of this study have important implications for both healthcare organizations and patient outcomes. A growing body of literature demonstrates the pivotal role of health literacy in achieving better health outcomes and higher quality of care ( Kaper *et al.*, 2019). Crucially, health literacy is modifiable and improving it is increasingly recognised as a way of enhancing outcomes, including in Europe’s Beating Cancer Plan ( EC, 2022). Shared decision-making and the use of interactive digital tools have been consistently associated with greater patient empowerment and improved results in cancer care ( Makarov *et al.*, 2021; Tuominen *et al.*, 2024a). In this context, preparing professionals to guide patients in the effective and secure use of digital tools becomes central to advancing patient-centred oncology.

The forthcoming European Health Data Space further underlines the need for robust digital skills among professionals, as health data will play a key role in research, innovation, and policy-making across Member States ( Marelli *et al.*, 2023). Therefore, it is essential that countries incorporate digital health literacy training not only into continuing education, but also at the earliest stages of health professional education—through university curricula, clinical rotations, and interdisciplinary learning opportunities. Supported by the dissemination capacity of scientific societies, universities, and European stakeholders, such efforts can accelerate the integration of digital health literacy into oncology care and strengthen workforce readiness.

At the health system level, addressing the digital divide—within and between countries—remains a prerequisite for reducing inequities in access to cancer care ( Saeed & Masters, 2021). By equipping professionals with the competencies needed to support patients with diverse digital skills, organizations can ensure that telemedicine, electronic health records, and other eHealth tools foster more equitable and patient-centred care. Ultimately, embedding digital health literacy into routine oncology practice not only enhances communication and clinical outcomes but also ensures that digitalisation contributes to narrowing—rather than widening—existing disparities across Europe.

### Strengths and limitations

This study has several strengths. First, it applied the NGT, a structured consensus method that minimises dominance effects and ensures equal participation, thereby enhancing the credibility and transparency of the findings. Second, the inclusion of both CP and NCP from diverse organisational settings and multiple European countries provided variability in professional profiles and geographical perspectives, which strengthens the transferability of the results. Third, conducting the workshops within the framework of the TRANSiTION project ensured that the identified priorities are aligned with a broader European effort to advance digital health literacy. Finally, to the best of our knowledge, this is one of the first studies to apply NGT to assess digital training needs in cancer care, representing an innovative approach that could serve as a methodological foundation for future research.

Nevertheless, some limitations should be acknowledged. The purposive sampling strategy, while appropriate for capturing relevant expertise, may limit the representativeness of the sample and the generalisability of the findings to other contexts or healthcare systems. In addition, some participants were members of the TRANSiTION consortium or had prior professional links with the research team, which may have influenced the workshop discussions, the interpretation of the focus question, or the prioritisation of recommendations. Although the structured NGT procedure, individual idea generation, moderated discussion, and anonymous voting were used to reduce this risk, the possible influence of these prior links cannot be fully excluded. Differences in workshop size, format, and the proportion of CP and NCP across workshops may also have influenced the emphasis placed on specific themes, limiting direct comparison between the two sessions. In addition, the workshops were conducted in English, which may have excluded professionals with limited language proficiency and thus narrowed the diversity of voices represented. Data analysis was primarily descriptive and relied on real-time consolidation of participant contributions rather than full transcript-based qualitative analysis, which may have reduced the depth of thematic exploration. Finally, the study was limited to two workshops, and no follow-up interviews or validation rounds were conducted to further refine or validate the recommendations.

## Conclusions

This study identified key priorities for advancing digital health literacy in oncology care through two NGT workshops with CP and NCP across Europe. These priorities include ethics, telemedicine, usability and user experience, equity, and continuous professional training, all of which are essential for integrating digital health into cancer care in a safe, effective, and patient-centred manner.

The findings reinforce calls from the scientific community and healthcare decision-makers to recognise digital skills as core healthcare competencies. Embedding these competencies into professional development frameworks has the potential to strengthen organisational capacity, support patient empowerment, and improve treatment outcomes. Future research should evaluate the implementation and impact of training initiatives derived from these priorities, ensuring that they remain responsive to evolving technologies, diverse patient needs, and the realities of oncology practice across Europe.

Ultimately, embedding digital health literacy into oncology care is not only a training challenge but also a strategic opportunity to build more equitable, patient-centred, and resilient health systems across Europe.

## Ethics and consent

The study was conducted within the TRANSiTION project and included two UX design workshops. For ethical oversight, we sought review from the Pontevedra–Vigo–Ourense Research Ethics Committee (ref: 2023/309), as this is the competent regional committee for non-pharmacological research activities under the Galician public health research system. The committee reviewed the study documentation and indicated that no formal evaluation was required for this workshop-based, minimal-risk qualitative activity.

Informed consent to participate was obtained verbally from all participants prior to the workshops. Written consent was not collected because the activities were conducted in a time-limited workshop format (hybrid and in-person) and involved minimal risk. Consent was documented through an explicit verbal consent procedure recorded at the start of each session, including consent for video recording for documentation and analysis purposes. Participants received an information sheet in advance and were informed that participation was voluntary and that they could withdraw at any time.

## Data Availability

Aggregated workshop data were generated and analysed during this study. All aggregated data supporting the findings are presented within the article, including prioritised recommendations, voting results, tables, and figures derived from the UX design workshops. Due to the group-based nature of data collection and the potential identifiability of participants through their individual contributions, raw participant-level inputs and full workshop records are not publicly shared. However, representative examples of participant contributions are included in Tables 1 and 2 to illustrate the range and content of the ideas generated.
