# Supplementary material for: Advancing digital health literacy in cancer care: Recommendations from two nominal group technique workshops in the TRANSiTION project
Source: Open Res Eur. 2026 May 21;6:22. Originally published 2026 Jan 12. [Version 4] doi: 10.12688/openreseurope.21439.4 (PMC13133620; doi:10.12688/openreseurope.21439.4)
Supplement: Peer review history

# Reviewer report: Reviewer response for version 3

**DOI:** 10.21956/openreseurope.25435.r72316

**Published:** 2026-04-30

**Authors:** Furqan Ahmed

**Affiliations:** 1 Department of Prevention and Evaluation, Leibniz Institute for Prevention Research and Epidemiology – BIPS, Bremen, Germany

This manuscript addresses an important topic and uses an appropriate Nominal Group Technique approach to identify digital health literacy priorities in cancer care. The article is relevant, clearly written, and provides practical recommendations.

 My comments are mainly about reporting clarity. In the Methods, Participants and Procedure sections, the authors should discuss more explicitly the possible influence of prior links between some participants and the TRANSiTION consortium on workshop discussion and prioritisation.

 In the Methods, Data analysis section, the manuscript should make even clearer that the thematic synthesis was conducted in real time to consolidate recommendations for voting and was not a full transcript-based qualitative thematic analysis.

 In the Results and Discussion, Main results sections, the comparison between the two workshops should be interpreted more cautiously because the workshops differed in size, format, and participant composition.

 In the Data availability statement, the wording should be revised to better reflect that aggregated workshop data were generated and analysed, even if raw data cannot be publicly shared due to identifiability concerns.

 Overall, I find the study useful and suitable for indexing.

Is the study design appropriate and does the work have academic merit?

Yes

Is the work clearly and accurately presented and does it cite the current literature?

Yes

If applicable, is the statistical analysis and its interpretation appropriate?

Not applicable

Are all the source data underlying the results available to ensure full reproducibility?

Partly

Are the conclusions drawn adequately supported by the results?

Yes

Are sufficient details of methods and analysis provided to allow replication by others?

Partly

Reviewer Expertise:

NCD, Cancer, Reproductive Health, Adolescent Health, AI in Prevention, Digital health

I confirm that I have read this submission and believe that I have an appropriate level of expertise to confirm that it is of an acceptable scientific standard, however I have significant reservations, as outlined above.

# Response

**Published:** 2026-05-06

**Authors:** David Liñares Mariñas

**Affiliations:** Instituto de Investigacion Sanitaria Galicia Sur, Vigo, Galicia, Spain

We thank the reviewer for their positive assessment of our manuscript and for the helpful suggestions to improve reporting clarity. We have revised the manuscript accordingly.

 First, we revised the Participants and Procedure sections to describe more explicitly that some participants were members of the TRANSiTION consortium or had prior professional links with the research team, whereas others were external invitees. We also clarified that these prior links may have made some participants more familiar with the project aims, terminology, and expected outputs before the workshops. In the Procedure section, we added information on how the structured NGT process, including silent individual idea generation, sequential sharing, moderated clarification, and anonymous voting, was used to reduce the potential influence of pre-existing relationships on group discussion and prioritisation. This issue has also been acknowledged in the Strengths and limitations section.

 Second, we revised the Data analysis section to clarify that participant contributions were consolidated in real time to prepare the list of recommendations for the voting phase. We now explicitly state that this process was part of the structured NGT procedure and was intended to support voting, rather than to produce a full qualitative thematic analysis. We also clarified that no formal coding tree, qualitative analysis software, post hoc transcript-based coding, or interpretive thematic analysis was conducted. Related wording in the Abstract, Procedure, Results, and Strengths and limitations sections has also been aligned by referring to real-time consolidation rather than thematic analysis or synthesis.

 Third, we revised the Results and Discussion, Main results sections to interpret the comparison between workshops more cautiously. We now state that the workshops differed not only in participant composition, but also in size and format. We have clarified that the observed differences should be understood as descriptive patterns within each workshop context, rather than as evidence of systematic differences between clinical and non-clinical professionals. This point has also been reflected in the Strengths and limitations section.

 Finally, we revised the Data availability statement to clarify that aggregated workshop data were generated and analysed during the study. We now specify that all aggregated data supporting the findings are presented within the article, including prioritised recommendations, voting results, tables, and figures. We also clarify that raw participant-level inputs and full workshop records are not publicly shared due to the group-based nature of data collection and the potential identifiability of participants through their individual contributions.

# Reviewer report: Reviewer response for version 2

**DOI:** 10.21956/openreseurope.25099.r71053

**Published:** 2026-03-18

**Authors:** Achraf ELOUERGHI

**Affiliations:** 1 Mohammed V University in Rabat, Rabat, Morocco

Summary of the Article

 This article reports on a qualitative study conducted within the European TRANSITION project, which aims to develop a comprehensive training framework to enhance digital health literacy among the oncology workforce. The study employed the Nominal Group Technique (NGT) within two user experience (UX) design workshops to identify and prioritize the digital health literacy training needs of both clinical professionals (e.g., oncologists, nurses) and non-clinical professionals (e.g., administrative, managerial staff) involved in cancer care across Europe.

 A total of 44 professionals from 16 countries participated. The first workshop (n=34) was predominantly composed of non-clinical professionals, while the second (n=10) mainly involved clinical professionals. Through a structured process of silent idea generation, group discussion, consolidation, and voting, five shared priorities emerged:

1. **Ethics:** Data protection, trust, and responsible use of digital tools.
2. **Telemedicine:** Integrating telemedicine to empower patients, provided it fosters safety and confidence.
3. **Usability and Accessibility:** Developing user-friendly, intuitive, and adaptable training interfaces.
4. **Equity and the Digital Divide:** Addressing the specific challenges faced by rural populations, older adults, and low-resource regions.
5. **Continuous Professional Training:** Establishing ongoing training with formal recognition of digital skills as core competencies.

The study also noted differences in emphasis between the two workshops: non-clinical professionals tended to prioritize technical design and telemedicine, while clinical professionals focused more on ethical considerations, patient trust, and the formal recognition of digital skills. The authors conclude that these findings provide an actionable foundation for the TRANSITION training framework and for broader European strategies to integrate digital health literacy into cancer care.

 1. Is the work clearly and accurately presented and does it cite the current literature?

**Yes.**

 The manuscript is exceptionally well-written, logically structured, and a pleasure to read. The introduction effectively sets the scene by outlining the challenges of digital transformation in oncology and the specific gap in workforce training. The discussion is comprehensive and demonstrates a strong command of the current literature. The authors successfully contextualize their findings within existing research on ethics in digital health (Bouderhem, 2024; Grosman-Rimon & Wegier, 2024), the double-edged nature of telemedicine (Petretto et al., 2024; Hur et al., 2022), the importance of user experience (Gilbert, 2022; Shamsujjoha et al., 2021), and the persistent challenge of the digital divide (Morris et al., 2022; Yuen et al., 2024). The citations are relevant, up-to-date, and appropriately support the arguments made.

 2. Is the study design appropriate and does the work have academic merit?

**Yes.**

 The study design is highly appropriate for the research aim. The Nominal Group Technique (NGT) is a well-established and robust method for generating and prioritizing consensus-based recommendations in healthcare research. Its structured approach minimizes the risk of dominant individuals skewing the results and ensures that all participants have an equal voice. Integrating NGT within a UX design workshop framework is a creative and effective approach that grounds the findings in practical, user-centered considerations. The study has clear academic merit, as it provides empirically derived, prioritized recommendations that directly address a significant gap in the literature and can inform the development of much-needed training frameworks and policies.

 3. Are sufficient details of methods and analysis provided to allow replication by others?

**Partly.**

*Strengths:* The procedural steps of the NGT workshops are clearly and thoroughly described. The authors detail the duration, format (hybrid vs. in-person), the central focus question, the process for silent idea generation, group discussion, consolidation, and the final voting phase. This level of detail allows for a clear understanding of how the workshops were conducted.

*Limitations and Recommendations for Improvement:*

1. **Lack of detail on purposive sampling criteria:** The authors state that participants were selected through "purposive sampling" by consortium members to ensure relevant expertise. However, the specific criteria used for this selection are not provided.
  - **Specific Suggestion:** In the "Participants" section, please add a brief description of the inclusion criteria. For example: "Participants were purposively sampled based on the following criteria: (a) current professional role in cancer care (clinical or non-clinical), (b) a minimum of [X] years of professional experience, and (c) involvement in or familiarity with digital health tools in their daily practice." This would clarify the sampling frame and allow for a more accurate assessment of the participant pool's expertise.
2. **Vague description of the "real-time thematic synthesis":** The methods section mentions that a researcher conducted a "real-time thematic analysis" or "synthesis" to group overlapping ideas for the voting phase. The process by which this was done is not explained.
  - **Specific Suggestion:** Please elaborate on this process. For instance: "During the group discussion, one researcher (initials) conducted an inductive, real-time synthesis of the ideas presented. Suggestions with similar meanings or concepts were grouped together on a virtual whiteboard. When ambiguity arose regarding the grouping of an idea, the researcher consulted the full group to confirm the appropriate categorization before finalizing the consolidated list for voting." This small addition would significantly improve the transparency of the synthesis process.

**These points should be addressed to make the article scientifically sound.**

 4. If applicable, is the statistical analysis and its interpretation appropriate?

**Not applicable.**

 The analysis is appropriately descriptive. The authors report the frequency and percentage of votes for each recommendation, which is the standard and correct approach for NGT studies. No inferential statistical tests are used, and the authors correctly refrain from overinterpreting the numerical results or making claims beyond the descriptive data. This is entirely appropriate for the study's aims.

 5. Are all the source data underlying the results available to ensure full reproducibility?

**Partly.**

*Strengths:* The authors state that "No additional datasets were generated" and that all data supporting the findings are included in the article. The aggregated results, including the prioritized recommendations and the voting percentages (displayed in Figures 1 and 2), are clearly presented.

*Limitations and Recommendations for Improvement:*

1. **Lack of raw data:** While the aggregated results are valuable, they represent the final, synthesized output of the workshops. A core principle of open science and reproducibility is to provide access to the underlying data that led to those results. In the context of an NGT study, this would be the complete list of ideas generated by participants *before* the consolidation and voting phase.
  - **Specific Suggestion:** To ensure full reproducibility and enhance transparency, the authors should provide the **complete, anonymized raw list of ideas generated by participants in each workshop** as supplementary material or in an online repository. This would allow readers to see the full breadth of input, understand the foundation upon which the consolidated recommendations were built, and verify the synthesis process. Anonymizing the list (e.g., "Participant 1, Workshop 1: ...") would protect participant privacy while providing invaluable data.

**This point should be addressed to make the article scientifically sound.**

 6. Are the conclusions drawn adequately supported by the results?

**Yes.**

 The conclusions are directly and logically derived from the results. The five shared priorities are a direct reflection of the voting outcomes and the synthesized themes from both workshops. The authors appropriately frame these priorities as actionable insights for the TRANSITION project and for broader European efforts to integrate digital health literacy into cancer care. The conclusions are well-calibrated to the scope of the study and do not overstate the findings.

Is the study design appropriate and does the work have academic merit?

Yes

Is the work clearly and accurately presented and does it cite the current literature?

Yes

If applicable, is the statistical analysis and its interpretation appropriate?

Not applicable

Are all the source data underlying the results available to ensure full reproducibility?

Partly

Are the conclusions drawn adequately supported by the results?

Yes

Are sufficient details of methods and analysis provided to allow replication by others?

Partly

Reviewer Expertise:

I am qualified to assess this manuscript based on my expertise in the following areas:- Health Services Research and Implementation Science: I have extensive experience in studying how evidence-based interventions, including training programs and digital health tools, are adopted and integrated into real-world healthcare settings. This allows me to evaluate the practical applicability and implementation potential of the recommendations presented in this study.- Qualitative and Mixed-Methods Research Methodology: I am proficient in a range of qualitative methodologies, including consensus methods such as the Nominal Group Technique (NGT) and Delphi method. I have both used and reviewed studies employing these techniques and can therefore assess the appropriateness, rigor, and reporting quality of the NGT application in this manuscript.- Health Workforce Development and Training: My expertise includes the design, delivery, and evaluation of continuing professional development programs for healthcare professionals. I can critically assess the study's findings regarding training needs, competency frameworks, and the proposed integration of digital skills into professional development pathways.- Digital Health Literacy and Patient-Clinician Communication: I am familiar with the literature on health literacy, digital health literacy, and the communication challenges that arise with the increasing use of technology in patient care. This enables me to evaluate the relevance and grounding of the study's identified priorities (ethics, telemedicine, usability, equity) within the broader field.

I confirm that I have read this submission and believe that I have an appropriate level of expertise to confirm that it is of an acceptable scientific standard, however I have significant reservations, as outlined above.

# Response

**Published:** 2026-03-23

**Authors:** David Liñares Mariñas

**Affiliations:** Instituto de Investigacion Sanitaria Galicia Sur, Vigo, Galicia, Spain

We thank the reviewer for the careful reading of our manuscript and for the constructive comments. We have addressed all methodological points raised and revised the manuscript to improve transparency and reproducibility.

**Comment 3. Are sufficient details of methods and analysis provided to allow replication by others?**

**Response:** We have clarified aspects of the sampling strategy and the real-time synthesis process. First, in the Participants section, we now specify the inclusion criteria for purposive sampling. Participants were required to be actively involved in cancer care, either in clinical or non-clinical roles, and currently engaged in their professional position. We also clarify that no minimum years of professional experience were required. Second, we expanded the description of the real-time thematic synthesis in the Data Analysis section. We now specify that overlapping suggestions were consolidated into unified statements, and that uncertainties in grouping or interpretation were resolved through consultation with participants. The final set of recommendations was agreed upon by the group before the voting phase, reflecting the structured and participatory nature of the Nominal Group Technique.

**Comment 5. Are all the source data underlying the results available to ensure full reproducibility?**

**Response: **All findings are presented in aggregated form within the manuscript, including prioritised recommendations, voting results, and representative examples of participant contributions (Tables 1 and 2). Due to the group-based nature of the workshops and the potential identifiability of participants, the full set of raw inputs is not publicly shared. This has been clarified in the Data Availability section. These revisions improve the clarity and transparency of the methodological description while remaining consistent with the study design and ethical considerations.

# Reviewer report: Reviewer response for version 2

**DOI:** 10.21956/openreseurope.25099.r70858

**Published:** 2026-03-18

**Authors:** Celine Jeitani

**Affiliations:** 1 Catholic University of Louvain, Louvain, Belgium

Adaptations seem ok.

Is the study design appropriate and does the work have academic merit?

Yes

Is the work clearly and accurately presented and does it cite the current literature?

Yes

If applicable, is the statistical analysis and its interpretation appropriate?

Not applicable

Are all the source data underlying the results available to ensure full reproducibility?

Partly

Are the conclusions drawn adequately supported by the results?

Yes

Are sufficient details of methods and analysis provided to allow replication by others?

Partly

Reviewer Expertise:

Health Literacy, Organizational Health Literacy, Psycho-oncology, Health Psychology (health behaviours).

I confirm that I have read this submission and believe that I have an appropriate level of expertise to confirm that it is of an acceptable scientific standard.

# Reviewer report: Reviewer response for version 1

**DOI:** 10.21956/openreseurope.23188.r68785

**Published:** 2026-02-14

**Authors:** Celine Jeitani

**Affiliations:** 1 Catholic University of Louvain, Louvain, Belgium

- Introduction is good, straight to the point and clearly shows the challenges faced.
- Methods section is okay. However, to a reader that has not followed the transition project, the framework behind the building of the workshop is not quite clear. The procedure is clear and aligns with the aim discussed in the introduction, but the part before the procedure can be developed to give a little bit more context on the framework that allowed for the procedure to come about.
- The data analysis basically means a collection and description of what was “written up” during the workshop. No “real” analysis was done. If that is the case, then the data analysis part is complete. If it isn’t, then it should be clarified.
- The results part needs to be further developed. The data given is interesting yet limited. The methodology touches upon “real-time thematic synthesis” that was used during the workshops, that could have been touched-upon in the results section portraying those themes. The methodology could be divided in subsections for each of the workshops as well to make it easier to follow and capture the importance of the “themes” emerged.
- The parallels between the results and scientific theory in the discussion is good. The authors show a good grasp of the literature. Some explained results in the discussion were further explained in the discussion vs the results section making it seem like the discussion has more “information” on results than the actual results section.
- The section on impact on organization can be integrated within the main discussion body before the limitations as it is a significant outcome interpretation of the study.

Is the study design appropriate and does the work have academic merit?

Yes

Is the work clearly and accurately presented and does it cite the current literature?

Yes

If applicable, is the statistical analysis and its interpretation appropriate?

Not applicable

Are all the source data underlying the results available to ensure full reproducibility?

Partly

Are the conclusions drawn adequately supported by the results?

Yes

Are sufficient details of methods and analysis provided to allow replication by others?

Partly

Reviewer Expertise:

Health Literacy, Organizational Health Literacy, Psycho-oncology, Health Psychology (health behaviours).

I confirm that I have read this submission and believe that I have an appropriate level of expertise to confirm that it is of an acceptable scientific standard, however I have significant reservations, as outlined above.
